# Effect of Sonic Activation on Push-Out Bond Strength of Fiber Post: An In Vitro Study

**DOI:** 10.3390/ma14175038

**Published:** 2021-09-03

**Authors:** Rizwan Jouhar

**Affiliations:** Department of Restorative Dentistry and Endodontics, College of Dentistry, King Faisal University, Al-Ahsa 31982, Saudi Arabia; rjouhar@kfu.edu.sa; Tel.: +966-59-3114621

**Keywords:** dental adhesives, sonic activation, push-out bond strength, self-adhesive resin cement, fiber post

## Abstract

This study aimed to evaluate the effectiveness of conventional and sonic activation techniques on push-out bond strength of fiber post cemented with two different monomers containing self-adhesive resin cement (SARC). Four groups (n = 19 each) were made based on the type of SARC (Rely X U200 and Panavia SA) and technique (conventional and sonic activation). After placing the fiber post, each root was sectioned into 2 mm coronal, middle, and apical portions, and a push-out bond strength test was performed using a universal testing machine. The least push-out bond strength (13.0 ± 0.9 MPa) was found in Rely X U200 conventional technique and highest with Panavia SA sonic activation technique (15.4 ± 0.9 MPa). A significant difference was found in push-out bond strength at coronal (*p* = 0.002), middle (*p* = 0.002), and apical (*p* = 0.001) root sections using Rely X U200 cement with sonic activation as compared to the conventional technique. However, no difference (*p* > 0.05) was noticed between conventional and sonic activation techniques in Panavia SA cement at any root level. Sonic activation can be used as an adjunct with a manual technique to increase bond strength. However, it was noted that 10-MDP monomer containing SARC performed well regardless of techniques.

## 1. Introduction

In the absence of sound coronal tooth structure, endodontically treated teeth are prone to failure due to fracture [1,2]. To reinforce coronally weak tooth structure, intraradicular post has been used for decades after endodontic treatment [3,4]. Both prefabricated and cast metal posts with various designs were utilized for a longer period in dentistry [5]. Despite their good intraradicular retention and presence of a thin layer of luting cement, the main drawbacks found in the metal post were poor esthetics and irreparable root fractures [3,5]. A higher modulus of elasticity of metal post as compared to root dentin was considered the main reason for root fractures [6,7].

To overcome these drawbacks, fiber-reinforced resin posts were introduced as an alternative, having high esthetic outcome, modulus of elasticity similar to root dentin, high flexural strength, and capability of bonding within the root dentin [3,8,9,10]. Fiber-reinforced post seats passively in the prepared root canal space, thus it relies mainly on adhesive cement for the retention [11]. The bond strength between root dentin and adhesive cement and also between fiber post and adhesive cement are the main factors for fiber post retention inside root canals [12,13,14,15]. Some clinical studies mentioned a 95% to 99% success rate of teeth restored with fiber-reinforced resin post with root fractures over a period of 2 years [16,17]. However, there are still failures associated with the use of fiber posts, most common of which include loss of retention due to debonding of adhesive cement [18,19,20,21]. Therefore, post cementation is a sensitive procedure as it involves several different aspects of dental adhesion such as bonding of fiber post to different substrates like dentin, cementum, and to restorative material [6,22].

Conventionally, a relatively technique sensitive multistep procedure was used for the placement of fiber post which included removal of smear layer by phosphoric acid etching, priming, and bonding followed by cementing the post with resin [23,24,25,26,27]. Later, self-etching primers were used to avoid etch and rinse procedures for better moisture control [18]. In the search of simplifying fiber post cementation technique, self-adhesive resin cement (SARC) were introduced [28,29]. Unlike previous resin cement, SARC is simple, time-saving, and has the advantage of fewer clinical steps required for the bonding procedure along with better control of moisture in the root canal space because no smear layer removal or pretreatment of dentin is required [30,31,32]. Functional acidic monomer in self-adhesive resin cement helps in bonding by having a strong affinity towards calcium in hydroxyapatite [29,33,34]. Studies have proven that phosphoric acid ester functional monomer 10-MDP in newer adhesives forms nano-layer at the adhesive-dentin interface that imparts better clinical longevity and integrity of the bond [35,36,37]. Also the interaction of monomer with hydroxyapatite results in the formation of monomer-calcium salts that are resistant to hydrolytic degradation and provide better adhesion with root dentin [38,39].

Failure in the formation of the interfacial layer due to inappropriate delivery of resin cement into the canal may lead to inadequate adhesion of post in the canal [40]. Centrix syringe systems are commonly used to overcome this problem; the device inserts the resin under pressure to achieve a better interfacial layer and adhesion of the post [41]. However, a deficient pressure may lead to inadequate adaptation of resin to dentin together with the formation of bubbles at the resin-dentin interface [33].

Studies have shown that ultrasonic and sonic activation of root canal irrigants, as well as root canal sealers, helps in their better penetration into canal walls and dentinal tubules [42,43,44,45,46]. Therefore, the active application of adhesives by sonic oscillating devices at 170 Hz may lead to better diffusion of material into root canal walls and lateral canals compared to the application with micro-brush [34,47,48,49]. Activation can increase their fluidity and the bubbles produced during their placement get pressed and burst against the canal walls [48,50]. In the light of the above, this study aims to evaluate the effectiveness of sonic activation on push-out bond strength of fiber post between two different monomers containing self-adhesive resin cement (SARC). The null hypothesis is that the sonic activation of self-adhesive resin cement does not improve the push-out bond strength of fiber-reinforced post regardless of the cement type.

## 2. Materials and Methods

### 2.1. Sample Preparation

The sample size was calculated using Open Epi sample size calculator by taking the mean bond strength 21.5 ± 3.9 MPa for group 1 (Panavia SA) and 17 ± 5.7 MPa for group 2 (Rely X U200), keeping a 95% confidence interval with a significance value set to *p* < 0.05 and power of test 80% [34]. The calculated sample size was 19 for each group. After the approval of the ethical review committee (KFU-REC/2020-10-19), seventy-six (19 × 4 groups) freshly extracted mandibular premolars were collected from the Oral Surgery Department of King Faisal University, Al Ahsa, Saudi Arabia. Only single-rooted teeth with single canal, mature apex, and extracted due to periodontal or orthodontic reasons were included. Whereas, teeth with curved roots, multiple roots, fracture lines, internal or external resorption, root caries, and immature apex were excluded from this study. Ligament residues, calculus, and soft debris were removed with ultrasonic scaler tips (UDS-J, Guilin Woodpecker, Guilin, China) and stored in 0.5% solution of chloramine water at 4 °C until employed in the experiment.

### 2.2. Post Space Preparation and Cementation

After collecting the required sample, teeth were decoronated with a diamond disk (IsoMet™1000; Buehler Ltd., Lake Bluff, IL, USA) at 1 mm above the cement-enamel junction, and a standardized length of 14 mm (±0.1 mm) was achieved. The canal was negotiated initially with manual K- File number 10, 15, and 20 (Dentsply, Maillefer, Switzerland) to make a smooth glide path, after which protaper universal file system (Dentsply, Maillefer, Switzerland) was used to shape the canal up to 1 mm short of working length till F3 file size. Throughout the instrumentation, 3% sodium hypochlorite was used as an irrigant. EDTA 17% irrigant (MD-Cleanser; Meta BIOMED) was used to remove the smear layer and then finally flushed with 3% sodium hypochlorite and distilled water. Roots were then dried with paper points and obturated with F3 gutta-percha and AH Plus (Dentsply, DeTrey) sealer. To allow the sealer setting, coronal 1 mm of the root was sealed with provisional restorative material (GC Fuji II, Tokyo, Japan), and teeth were then stored in 100% humidity at 37 °C for 48 h. Subsequently, the precision drill was used to prepare space for Rely X fiber post (3M ESPE, Deutschland GmbH, Neuss, Germany) till size 3 up to the length of 10 mm from the cementoenamel junction (overall 11 mm). A new drill set was used to prepare post space after every five specimens. The post space was then irrigated with saline and dried with the paper point. Size 3 fiber post was placed inside the canal to check its seating and length achievement by marking it at 11 mm. After making sure that the post is completely seated at 11 mm length (including 1 mm above the cement-enamel junction) it was removed and cut to the marked length with a diamond disk (IsoMet™1000; Buehler Ltd., Lake Bluff, IL, USA). Posts were then cleaned with 70% alcohol for 30 s, rinsed with distilled water, air-dried, and stored in a sterilized pouch until fiber post cementation was performed with the SARC (Table 1). All procedures were performed by the principal investigator.

The root specimens and prepared fiber posts were randomly divided into four groups:

**Group 1:** Rely X U200 Cement, conventional (n = 19)

Rely X U200, Self-adhesive resin cement (3M ESPE, Deutschland GmbH, Neuss, Germany) was inserted into the post space with centrixautomix tip with forceful injection until it oozed out of the canal and then fiber post was placed into the canal till it seated completely up to the prepared length. Excess cement was removed and light-cured (standard power: 100 mW/cm^2^) for 40 s from the coronal position.

**Group 2:** Rely X U200, sonic activation (n = 19)

Rely X U200, Self-adhesive resin cement (3M ESPE, Deutschland GmbH, Neuss, Germany) was inserted into the post space with centrixautomix tip and then it was sonically activated with an endoactivator (Dentsply, Maillefer, Ballaigues, Switzerland) for 10 s at the frequency of 170 Hz. The remaining space after endoactivator tip removal was then filled and the fiber post was placed into the canal till it was seated completely up to the prepared length. Excess cement was removed and light-cured (standard power: 100 mW/cm^2^) for 40 s from the coronal position.

**Group 3:** Panavia SA, conventional (n = 19)

Panavia SA, self-adhesive resin cement (Kuraray, Tokyo, Japan) was inserted into the post space with centrixautomix tip with forceful injection until it oozed out of the canal and then fiber post was placed into the canal till it seated completely up to the prepared length. Excess cement was removed and light-cured (standard power: 100 mW/cm^2^) for 40 s from the coronal position.

**Group 4:** Panavia SA, sonic activation (n = 19)

Panavia SA, self-adhesive resin cement (Kuraray, Tokyo, Japan) was inserted into the post space with centrixautomix tip and then it was sonically activated with an endoactivator (Dentsply, Maillefer, Switzerland) for 10 s at the frequency of 170 Hz. The remaining space after endoactivator tip removal was then filled and the fiber post was placed into the canal till it was seated completely up to the prepared length. Excess cement was removed and light-cured (standard power: 100 mW/cm^2^) for 40 s from the coronal position.

### 2.3. Push-Out Bond Strength Test

**Figure 1:**
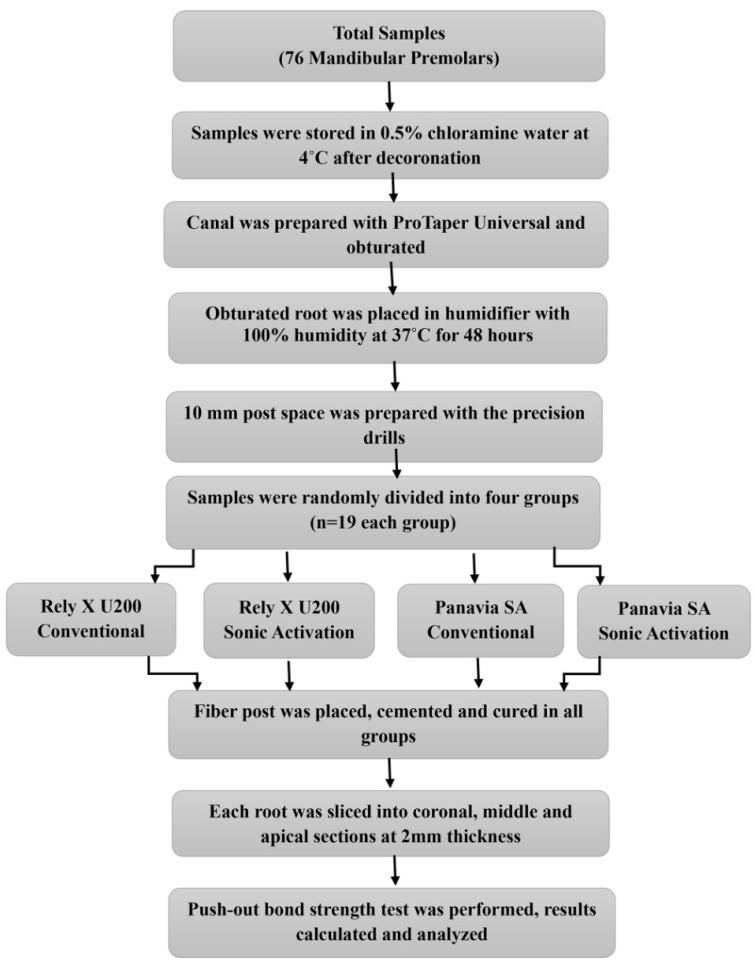
Flowchart of the methodology.

**Figure 2:**
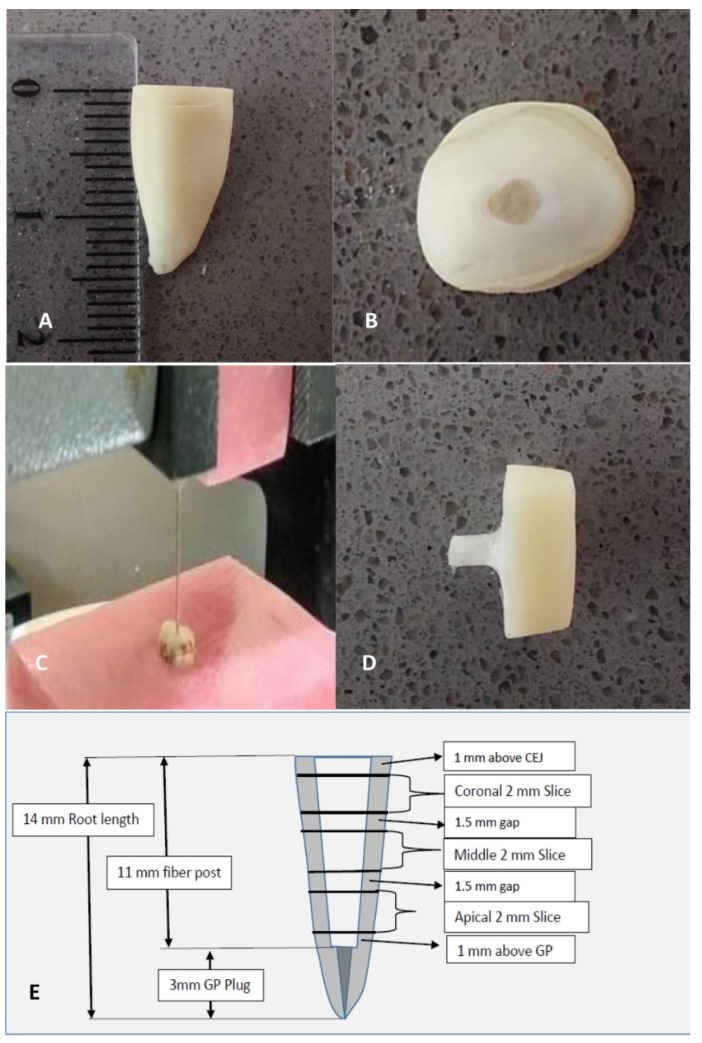
Pictorial illustration of the methodology. (**A**): 14 mm sectioned root; (**B**) Fiber post placed; (**C**) Push-out test performed using Universal Testing Machine; (**D**) Push-out fiber post from the root section; (**E**) Schematic illustration of root sectioning.

Coronal, middle, and apical slices of 2 mm thickness were obtained from each specimen by cutting them with a low-speed diamond disk (IsoMet™1000; Buehler Ltd. Illinois, USA) under constant water-cooling at 200 rpm (Figure 1 and Figure 2). These slices were tested for push-out bond strength using the universal testing machine (Instron 4301, Canton, MA, USA) at speed of 1 mm/min until the bond failure and extrusion of the post. The loading rod diameter of the push-out testing machine was kept 0.2 mm shorter than the diameter of the specimen. The large radius ‘R’, small radius ‘r’, and height for each specimen were calculated by using a digital caliper (Mitutoyo, Suzano, Brazil) which has a range of 0 to 150 mm. The universal testing machine indicated the force required for bond failure in Newton (N) which was then converted into mega Pascal (MPa) by dividing it with the area of the bonded surface. Area of dentin-post interface was calculated with formula A = π (R + r) [h^2^ + (R − r)^2^]^0.5^, where ‘π’ is equal to 3.14, ‘R’ is the greatest radius and ‘r’ is the smallest radius taken from coronal and apical aspects of each slice, ‘h’ represents slice height.

Push-out bond strength was calculated in MPa for each individual group at the three root levels (coronal, middle, apical). Collected data were analyzed by using statistical software SPSS, Version 25.0. (IBM Corp., Armonk, NY, USA). The mean and standard deviation of bond strength was calculated and one-way ANOVA was used to assess significance among bond strength of each group at different root levels. Comparison of bond strength by two techniques within a cement was done using paired *t*-test. A *p*-value of ≤0.05 was considered to be significant. Differences in mean bond strengths of different types of cement at coronal, middle, and apical levels were assessed by using an independent *t*-test.

## 3. Results

The mean and standard deviation of push-out bond strength of each root section among four groups was calculated and described in Table 2. The least total mean push-out bond strength (13.0 ± 0.9 MPa) was found in Rely X U200 conventional technique as compared to Panavia sonic activation technique which achieved a maximum bond strength (15.4 ± 0.9 MPa) while Rely-X with sonic activation and Panavia SA conventional techniques showed an intermediate bond strength of 14.2 ± 0.7 MPa and 14.9 ± 0.5 MPa respectively. In addition, no significance difference was found in the bond strength at different root levels for each of the SARC in the tested techniques.

**Figure 3:**
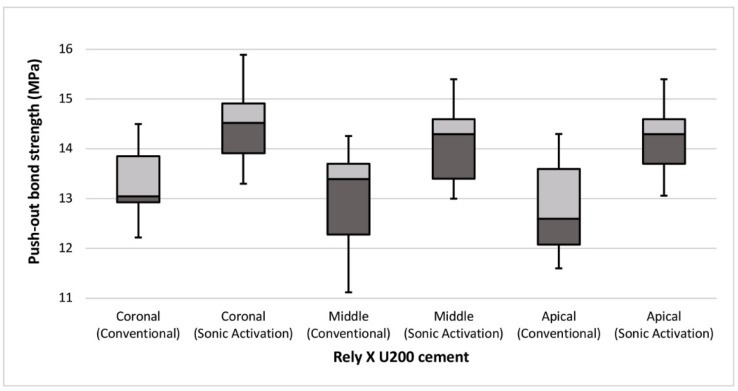
Comparison of mean bond strength between conventional and sonic activation at different root levels in Rely X U200 SARC.

**Figure 4:**
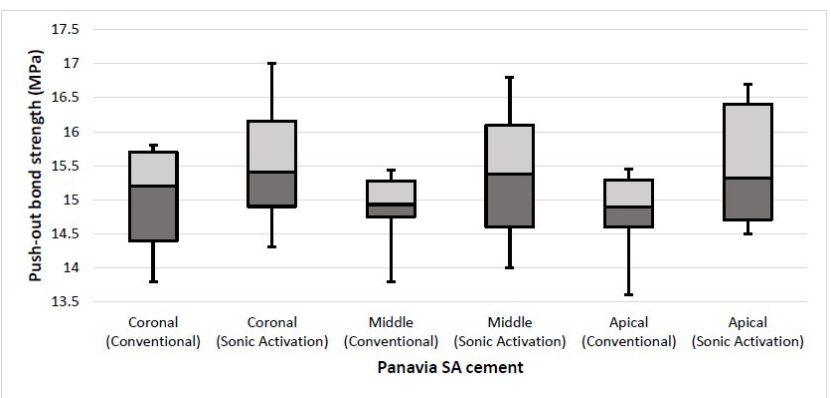
Comparison of mean bond strength between conventional and sonic activation at different root levels in Panavia SA SARC.

On comparing the two techniques, a significant difference (*p* ≤ 0.05 considered significant) was found in the coronal (*p* = 0.002), middle (*p* = 0.002), and apical (*p* = 0.001) mean bond strengths with Rely X U200 cement. The push-out bond strength was improved when sonic activation was applied with Rely-X U200 cement (Figure 3). However, the bond strength with Panavia SA cement was not affected in both techniques. No significant differences (*p* > 0.05) were found in mean bond strengths at coronal (*p* = 0.164), middle (*p* = 0.315), and apical (*p* = 0.146) portions of the roots between conventional and sonic activation techniques (Figure 4). 

Furthermore, a comparison between Rely X U200 and Panavia SA was performed at different root levels (Table 3). A significant difference was found at coronal, middle, and apical root portions (*p* ≤ 0.001) when mean bond strengths of the two types of cement were compared using the conventional technique. Moreover, the result was also significant (*p* ≤ 0.005) when the mean bond strengths of two types of cement were compared using the sonic activation technique at different root levels, with increased values of bond strength for Panavia SA cement regardless of the technique used.

## 4. Discussion

The current study was perfomed to evaluate the effectiveness of conventional and sonic activation techniques on push-out bond strength of fiber reinforced composite (FRC) post luted with two different monomers containing self-adhesive resin cement (SARC). The results of the present study showed that fiber post cemented directly with the conventional technique (centrix syringe system) did not show any significant difference (*p* > 0.05) in the push-out bond strength compared to the sonic activation technique in Panavia SA SARC. Conversely, a significant difference (*p* < 0.05) was found with sonic activation of Rely X U200 SARC as compared to the control group. Hence, the null hypothesis was partly rejected.

The push-out bond strength test is performed to assess retention of bonded FRC posts in the root canal, while at the same time, it also allows microscopic analysis of marginal adaptation of cement and level of bond failure [51]. It also evaluates the uniform stress distribution throughout the resin-dentin interface and minimises the chances of premature failure. The good result of push-out bond strength under sonic activation may be due to better cement flow, reduced viscosity, increase in degree of conversion of the material and decreased bubble formation [34].

The result of current study is in line with a previous study in which push-out bond strength with or without sonic activation in bovine incisors remained non-significant when luted with Panavia SA cement, while a significant increase in push-out bond strength was found in Rely X U200 cement with sonic activation [34]. Moreover, in the same study authors reported a significant difference in bond strength with sonic activation of Bifix SE cement (Voco GmbH, Cuxhaven, Germany) and no difference in set PP cement (SDI, Bayswater, Australia) as compared to the control group [34]. This may be due to the presence of 10-MDP monomer in Panavia SA as compared to methacrylate monomer (MMA) in Rely X U200 and Bifix SE.

Panavia SA SARC contains a 10-MDP monomer, which has a higher affinity for calcium, is resistant against hydrolysis, and plays a major role in cement to hydroxyapatite interaction [52]. Studies have shown that phosphoric acid ester functional monomer 10-MDP in newer adhesives which forms a nano-layer at the adhesive-dentin interface that imparts better clinical longevity and integrity of the bond [35,36,37]. In addition, this monomer makes a very stable bond with the calcium releases from the dissolution of hydroxyapatite crystals [53]. This chemical affinity is responsible for the low rate of dissolution of calcium salts, resulting in better performance of these materials in push-out bond strength [54]. This may be the reason for the higher bond strength obtained in Panavia SA SARC compared to Rely X U200 SARC, despite the use of different techniques. Moreover, no significant effect of technique was established while using Panavia SA SARC.

Rely X U200 SARC contains phosphoric acid monomer and modified multifunctional methacrylate monomer. In our study, Rely X U200 bond strength was significantly increased with the sonic activation technique. The increased in bond strength may be due to the fact that the centrix system alone does not exert enough pressure, while sonic activation is believed to promote wettability and interaction of cement to the dentinal walls. On contrary, Keter et al. have reported a negative effect of ultrasonic and sonic activation on push-out bond strength of fiber post when luted with Rely X U200 [21]. This difference may be due to the increased in temperature within the canal after the use of ultrasonic or sonic activation which interns expedite polymerization shrinkage and eventually exert high stresses on the canal walls. Moreover, differences in methodology and sample size may also contribute to this contradict results. Although sonic or ultrasonic activation of irrigants can increase temperature [55], no study reported temperature rise of resin by such activation inside the canal.

The results of current study must be seen with certain limitations such as the sample did not undergo aging or mechanical cycling, the non-availability of scanning electron microscopy (SEM) to evaluate level of bond failure at cement-dentin-post interface. Furthermore, the tensile strength test was not performed hence the stress-strain curve could not be extrapolated. Lastly, only premolars with round-shaped canals were included in this study, hence future studies are desirable to evaluate the effect of sonic activation on oval-shaped canals. In addition, a clinical trial with a large sample size will also help to establish the effect of sonic activation on the clinical performance of the material.

The author of the current study could not find any research showing a negative or positive effect of sonic activation on push-out bond strength by comparing, Rely X U200 and Panavia SA cements in human teeth. The results may help clinicians by taking advantage of sonic activation when using Rely X U200 cement to increase the clinical success of endodontic cases requiring fiber posts.

## 5. Conclusions

Within the limitations of this in-vitro study, it could be concluded that a 10-MPD monomer containing SARC can provide equally good results regardless of the technique used. However, with the use of sonic activation, the bond strength of the methacrylate monomer (MMA) containing SARC can be increased significantly. Thus this technique can be used as an adjunct to the manual placement of the cement.

## Figures and Tables

**Table 1:** Composition of self-adhesive resin cement.

**Rely X U200 Cement**	**Base paste:** Silanated glass powder, 2-Propenoic acid, 2-methyl,1,10-[1-(hydroxymethyl)-1,2-ethanodiyl] ester, triethylene glycol dimethacrylate (TEGDMA), silane-treated silica, glass fiber, sodium persulfate, and Tert-butyl peroxy-3,5,5-trimethylhexanoate. **Catalyst paste:** Silanated fillers, dimethacrylate substitute, Sodium p-toluenesulfonate, 1-Benzyl-5-phenylbarbituric acid, calcium salts, 1,12-Dodecanediol dimethacrylate, calcium hydroxide, and titanium dioxide.
**Panavia SA Cement**	**Base paste:** 10-Methacryloyloxydecyl dihydrogen phosphate (MDP), bisphenol A diglycidyl methacrylate (Bis-GMA), Triethylene Glycol Dimethacrylate (TEGDMA), hydrophobic dimethacrylate, 2 hydroxymethylmethacrylate (HEMA), silanated barium, silanated colloidal silica, dl-Camphorquinone, peroxide, catalyst, and pigments. **Catalyst paste:** Hydrophobic aromatic dimethacrylate, hydrophobic aliphatic dimethacrylate, silinated barium, sodium fluoride, accelerators, and pigments

**Table 2:** Mean and Standard Deviation of different techniques at root levels.

Techniques and Root Levels		Mean (MPa)	SD (MPa)	*p*-Value
Rely X U200 Conventional	Coronal	13.3	0.8	0.266
	Middle	12.9	1.0	
	Apical	12.8	0.9	
	**Total**	**13.0**	**0.9**	
Rely X U200 Sonic Activation	Coronal	14.4	0.8	0.487
	Middle	14.1	0.7	
	Apical	14.1	0.7	
	**Total**	**14.2**	**0.7**	
Panavia SA Conventional	Coronal	15.0	0.7	0.708
	Middle	14.9	0.5	
	Apical	14.8	0.5	
	**Total (n = 19)**	**14.9**	**0.5**	
Panavia SA Sonic Activation	Coronal	15.6	0.9	0.658
	Middle	15.3	0.8	
	Apical	15.4	0.9	
	**Total (n = 19)**	**15.4**	**0.9**	

**Table 3:** Mean comparison of Panavia and Rely X U200 cements between techniques.

Comparison between Conventional Techniques		Coronal	Middle	Apical
Rely X U200 vs. Panavia SA	*Mean difference*	−1.6	−1.9	−2.0
	*Standard error*	0.3	0.3	0.3
	*t*	−4.6	−5.1	−5.4
	*p-value*	0.000	0.000	0.000
	*95% confidence interval* *Lower* *Upper*	−2.4 −0.9	−2.7 −1.1	−2.7 −1.2
**Comparison between Sonic Activation Techniques**		**Coronal**	**Middle**	**Apical**
Rely X U200 vs. Panavia SA	*Mean difference*	−1.1	−1.1	−1.3
	*Standard error*	0.4	0.3	0.3
	*t*	−2.7	−2.8	−3.3
	*p-value*	0.014	0.011	0.004
	*95% confidence interval* *Lower* *Upper*	−2.0 −0.2	−1.9 −0.3	−2.1 −0.4

(*t*; variation in mean value).

## Data Availability

The study data is available from the corresponding author on request.
